# Whole-genome resequencing analysis of the medicinal plant *Gardenia jasminoides*

**DOI:** 10.7717/peerj.16056

**Published:** 2023-09-18

**Authors:** Xinyu Xu, Bihua Chen, Juan Zhang, Siren Lan, Shasha Wu

**Affiliations:** 1 Fujian Academy of Forestry Sciences, Fuzhou, Fujian, China; 2 College of Landscape and Architecture, Fujian Agriculture and Forestry University, Fuzhou, Fujian, China

**Keywords:** *Gardenia jasminoides*, Whole genome-resequencing, SNP, InDel, SV, CNV, Crocin, Geniposide

## Abstract

**Background:**

*Gardenia jasminoides* is a species of Chinese medicinal plant, which has high medicinal and economic value and rich genetic diversity, but the study on its genetic diversity is far not enough.

**Methods:**

In this study, one wild and one cultivated gardenia materials were resequenced using *Illumina*HiSeq sequencing platform and the data were evaluated to understand the genomic characteristics of *G. jasminoide*s.

**Results:**

After data analysis, the results showed that clean data of 11.77G, Q30 reached 90.96%. The average comparison rate between the sample and reference genome was 96.08%, the average coverage depth was 15X, and the genome coverage was 85.93%. The SNPs of FD and YP1 were identified, and 3,087,176 and 3,241,416 SNPs were developed, respectively. In addition, SNP non-synonymous mutation, InDel mutation, SV mutation and CNV mutation were also detected between the sample and the reference genome, and KEGG, GO and COG database annotations were made for genes with DNA level variation. The structural gene variation in the biosynthetic pathway of crocin and gardenia, the main medicinal substance of *G. jasminoides* was further explored, which provided basic data for molecular breeding and genetic diversity of *G. jasminoide*s in the future.

## Introduction

*Gardenia jasminoides* Ellis (2n = 22) is an evergreen shrub of the genus *Gardenia* in the Rubiaceae family. It is distributed in many areas of China and is mainly used in the cultivation of medicinal plants and garden landscape. *G. jasminoides* is known for its special aroma and is an excellent greening tree (Xiao et al., 2017). Gardenia has very high medicinal value, and its fruits, leaves, flowers and roots can be used as medicine. It is an ideal Chinese medicinal plant (Zhao et al., 2017; Lu et al., 2019; Zhang, Bian & Yao, 2022). The chemical components of gardenia fruits are mainly iridoids, including geniposide and gardenic acid, among which geniposide is one of the most active components (Long et al., 2013; Xu et al., 2016; Zhou et al., 2021). In addition, gardenia also contains carotenoid compounds, such as crocin, which can regulate the neural center, improve memory and cognition (Tian et al., 2020; Shen et al., 2022), and phenolic acid compounds, mainly chlorogenic acid, which can lower blood pressure, antioxidant, free radical scavenging and regulate the body’s immunity (Liu et al., 2020). With the continuous research, development and utilization, gardenia has become the first batch of edible and medicinal plants in China. Gardenia yellow can be used as natural food colorant and food coloring agent (Wu et al., 2022). In Thailand, *G. jasminoides* is used in religious rituals and hair decorations, and in other Asian countries, *G. jasminoides* is even used in cooking (Chen et al., 2020a; Chen et al., 2020b).

In recent years, whole genome resequencing has been conducted on many plants, such as soybean, rice and sorghum (Habyarimana et al., 2022; Long et al., 2022; Wang et al., 2022). In order to detect various types of genetic variation and improve and domesticate genes better, whole genome sequencing on 292 pieces of pigeonpea were conducted and found several genome regions that might be targets for domestication and breeding. Then, through genome-wide association analysis, it was found that the genes related to flowering time control, seed development and pod cracking had sequence similarities with other plants. Meanwhile, some trait selection areas were consistent with geographical distribution, suggesting that different geographical environments led to the adaptive changes of some traits in pigeonpea (Varshney et al., 2017). On the other hand, some studies have provided a new perspective for the evolution of loquat genome and fruit domestication by resequencing wild loquat (Jing et al., 2023). In addition, resequencing analysis and KEGG enrichment analysis were used to identify differences in nitrite metabolism between ’Linhuang No.1′and ’Muzao’ cultivars (Li et al., 2021). Genomes including heavy single nucleotide polymorphisms (SNPs) and insertions or deletions (indel) through high-throughput were sequenced to detect complex genetic variation, for example, structure variation (SV) and copy number variation (CNV) (Ganal, Altmann & Röder, 2009; Adedze et al., 2021; Ma et al., 2021). A total of 520,260 SNP variations were identified after sequencing the whole genome of 18 flue-cured virginia (FCV) tobaccos (*Nicotiana tabacum*). A total of 4,849 homozygotes and 28,584 polynucleotide polymorphisms were also detected, which are critical for the development of excellent tobacco breeding lines (Thimmegowda et al., 2018). InDel markers based on whole genome resequencing have attracted more and more attention due to their advantages of wide distribution, high density, stable variation, strong polymorphism and easy detection in the genome. For example, different InDel markers have been developed based on resequencing data in terms of watermelon peel traits and color (Li et al., 2018). InDel markers were used to identify genetic loci related to the regulation of peanut bacterial wilt resistance genes (Zhang et al., 2022). From the development of gardenia genome, it was found that the functional genes involved through tandem gene replication in gardenia fruit (Xu et al., 2020). At present, there are few reports on genetic diversity analysis of germplasm resources of gardenia, and due to the differences or incomplete species of the gardenia population selected by various researchers. There are abundant variation conditions among wild populations, the polymorphism between and within the species is not completely consistent. The previous study results can not totally reflect the genomic characteristics of these genetic resources. At the same time, due to the lack of genomic data, there are few stable and reliable molecular markers, which greatly limits the progress of genetic breeding of gardenia.

In this study, we employed whole-genome resequencing technology to align the genomes of one wild types and one cultivated variety of *G. jasminoides* to the reference genome of *G. jasminoides*. Based on this, we conducted differential analysis at both individual and population levels, aiming to explore the differences and structural variations in gene sequences. This analysis included InDel detection and annotation, SV detection and annotation, distribution of all variations on the genome, and analysis of genes involved in the variation of geniposide and crocin production. Functional enrichment analysis was performed to annotate the variant genes, laying the foundation for the genetic study of *G. jasminoides*.

## Materials and Methods

### Sample collection, DNA extraction and sequencing

The sample of *G. jasminoides* (FD) used for genome resequencing analysis is an improved variety cultivated in Fuding City, Fujian Province, it was selected by the fruit indexes of different cultivated varieties, and then obtained by asexual propagation. Gardenia YP1 is a wild *G. jasminoides* in Yanping, Fujian Province. Twenty fresh leaves of gardenia FD and 20 fresh leaves of gardenia YP1 were collected at flowering stage, and genomic DNA was extracted from young leaves by cetyltrimethyl ammonium bromide (CTAB) (Madhukar et al., 2020). The concentration and quality of total genomic DNA were determined by NanoDrop 2000 spectrophotometer (Thermo Fisher Science, Waltham, MA, USA), and the DNA library of *Illumina*/BGI sequencing (350 bp) was constructed. After the library was constructed, the library was sequenced on the *Illumina* HiSeq X Ten/Nova Seq/BGI platform and read as 150 bp. Finally, high-quality sequences are obtained for follow-up analysis.

### Tools for variation analysis

The detection of SNP and small InDel is mainly realized by GATK (v4.1) (Lin et al., 2022) software kit. Use SAMtools (v1.9) (Danecek et al., 2021) to filter redundant reads to ensure the accuracy of the test results. Then the local haplotype assembly (Haplotype Caller) algorithm of GATK is used to detect the mutation of SNP and InDel. Each sample first generates gVCF, and then carries on the population joint-genotype. In order to ensure the reliability of the mutation result, based on the subroutine vcfutils. pl (varFilter-w 5-W 10) in BCFtools, SNPs within 5bp of an InDel and within 10bp if there is an adjacent InDel are filtered out. The filtering parameters are QUAL < 30, QD < 2.0, MQ < 40, feeds FS > 60.0. Other variation filtering parameters are processed by the default values officially specified by GATK, and the final set of variation sites is obtained. SV mutation detection uses Manta (v1.6) (Kosugi et al., 2019) software to detect insertion (INS), deletion (DEL), inversion (INV), chromosome translocation (TRA) between sample and reference genome based on the relationship between pair-end reads alignment and reference genome and actual insert size. CNV detection uses FREEC (Wei, Dugas & Sandmann, 2021) to detect the depth distribution of reads on the reference genome by sample sequencing.

### Alignment of reference genomes

The original reads (double-segment sequence) was analyzed by de-splicing and low-quality reads filtration analysis, and finally clean reads were obtained for mutation gene detection. The clean reads of FD (GenBank BioSample: SAMN35881984) and YP1 (GenBank BioSample: SAMN35881985) of gardenia were compared with the reference genome. (https://www.ncbi.nlm.nih.gov/genome/?term=Gardenia_jasminoides, *i.e.,* GenBank: assembly ASM1310374v1) (Xu et al., 2020). TBtools (Chen et al., 2020) was used to search for the required variant gene information in the reference genome, and then compared and analyzed on NCBI.

### Functional annotation of the variant gene of GO, COG, KEGG

Through the comparison of the reference genome, BLAST compared the mutant genes with the functional database such as GO, COG, KEGG (Altschul et al., 1997; Kanehisa et al., 2004; Gene Ontology Consortium, 2015) to obtain the annotations of these genes to analyze the gene function. The screening criteria for enrichment analysis are P- ≤ 10^−5^ and false discovery rate (FDR) ≤ 0.01, respectively.

## Results

### Analysis of the results compared with the reference genome

In this study, a total of 534.9 Mb were mapped to the reference genome (Xu et al., 2020). The sequencing data of the database were filtered after quality control. The average value of each sample was about 39 million clean reads, 11.77 Gbp clean data. For gardenia FD and YP1, Q20 was 96.86%, 96.49% and Q30 was 91.34%, 90.58%, respectively (Table 1), and the average coverage depth of each sample was 10x. Compared with the reference genome, the percentage of clean reads mapped to the reference genome by FD is 95.57%. The comparison result is good (Table 2), and the proportion of genome coverage corresponding to each depth of FD (1x, 5x, 10x) is 85.02%, 71.13%, 46.02%, YP1 is 86.84%, 67.26%, 35.96% (Table S1). According to the coverage depth of each point of the chromosome, it was found that the coverage depth was evenly distributed on the chromosome (Figs. S1a, S1b), indicating that the genome was evenly covered and the sequencing was random.

**Table 1:** Evaluation statistics of sample sequencing data.

**Sample ID**	**Clean Reads**	**Clean Base**	**Q20 (%)**	**Q30 (%)**	**GC (%)**
FD	21,292,407	6,361,513,792	96.86	91.34	36.85
YP1	18,114,168	5,408,207,524	96.49	90.58	36.96

**Table 2:** Statistics of comparison results with reference genome.

**Sample ID**	**Total reads**	**Mapped (%)**	**Properly mapped (%)**
FD	42,584,814	95.57	83.05
YP1	36,228,336	96.59	87.03

### Mutation detection and annotation

**Figure 1:**
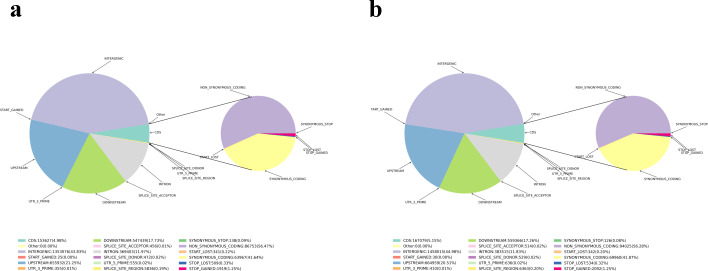
InDel comment result statistical chart. (A) FD of InDel comment result statistics. (B) YP1 of InDel comment result statistics. The light blue part indicates CDS, the dark blue part indicates upstream, the purple part indicates intergenic, the gray part indicates intron, the yellow part indicates synonymous coding , the red part indicates the start gained (non-coding region), and the navy blue part indicates the start lost.

**Figure 2:**
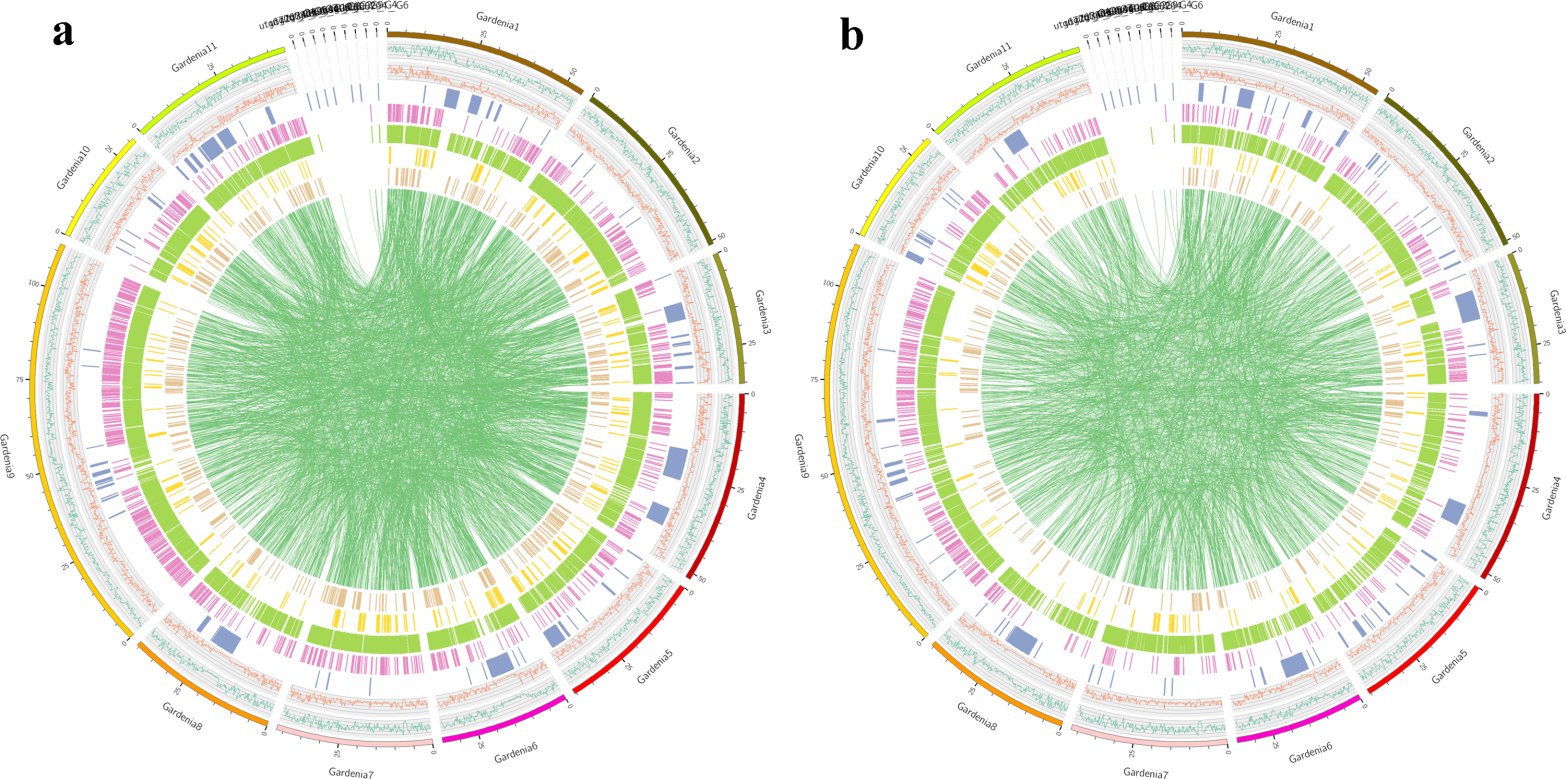
Distribution of various types of variation in chromosomes of samples. (A) Distribution of various types of variation in chromosomes of FD. (B) Distribution of various types of variation in chromosomes of YP1. From outside to inside, the order is: chromosome coordinates, SNP density distribution, InDel density distribution, CNV, SV [INS, DEL, INV, DUP, TRA] distribution on the genome (unit M).

According to the mapping results of clean reads in the reference genome, the SNP, InDel, SV and CNV mutations of two samples FD and YP1 were detected. For SNP mutation, 3,087,176 and 3,241,416 high quality SNP sites were obtained respectively. Then the SNP was annotated, in which the sample FD conversion and subversion ratio was 1.87, the sample YP1 conversion and subversion ratio was 1.89, and the heterozygous SNP ratio FD was 49.64% and 67.07%. The heterozygosity of sample FD is lower than that of sample YP1 (Table S2). From the Venn map between samples, it was found that there were 1,737,580 SNP variants in FD and YP1, 1,503,846 SNP variants specific to YP1, and 1,349,606 in FD (Fig. S2). The SNP mutations of the whole genome can be divided into six types, among which the number of T:A > C:G in YP1 is more than that in FD (Fig. S3). The results of SNP annotation showed that there was no significant difference in SNP variation between FD and YP1 (Figs. 1A, 1B). After that, the InDels of small fragments between the sample and the reference genome were detected. The numbers of InDels of FD CDS and genome fragments were 10,270 and 558,766, the YP1 is 10,752 and 540,581. the number of InDels in the coding region of YP1 was more than that of FD (Fig. S4), and the number of homozygous InDel of FD in the whole genome and coding region was higher than that of YP1, and the number of YP1 heterozygous InDel in the whole genome and coding region was higher than that of FD (Table S3, Figs. 2A, 2B). In addition, the genomic SV of the two samples was detected and annotated. There were 11,543 and 7,634 SV variants in FD and YP1. The number of insertion type variation, deletion type variation, inversion type variation, repetition type variation and chromosome translocation type variation in FD were higher than those in YP1. The annotation results of SV showed that the variation of FD in the gene was more than that in the intergenic region, and the insertion and deletion in the intergenic region was much higher than that in the gene, while the reversal and repetition were much lower than those in the gene. In the annotation of CNV, 885 variant genes, 956 lost genes, 840 predicted single copy number, 885 variant genes, 780 deletion genes and 770 predicted single copy number were obtained by FD substitution type and YP1 substitution type, respectively. The number of variation obtained by FD and YP1 was the same, and the number of lost genes was more than YP1, and the number of single copy was also more than that of YP1 (Table S4).

Variation in the CDS region may cause changes in gene function. By looking for genes with non-synonymous mutations in SNP, InDel and SV in the CDS region between the reference genome and the sample, the genes were found that may have functional differences between the sample and the reference genome. Among the differential gene variations in FD, the type with the largest number of mutations was SNP, with a total of 23,338 variations. In YP1, the number of differential genes was also the most in SNP, with 24,631, in which the number of InDel mutants in YP1 was more than that in FD, while the number of SV variations was on the contrary, and SV variations in FD were more than YP1 (Table 3, Fig. 1).

### Functional annotation at the DNA level

**Figure 3:**
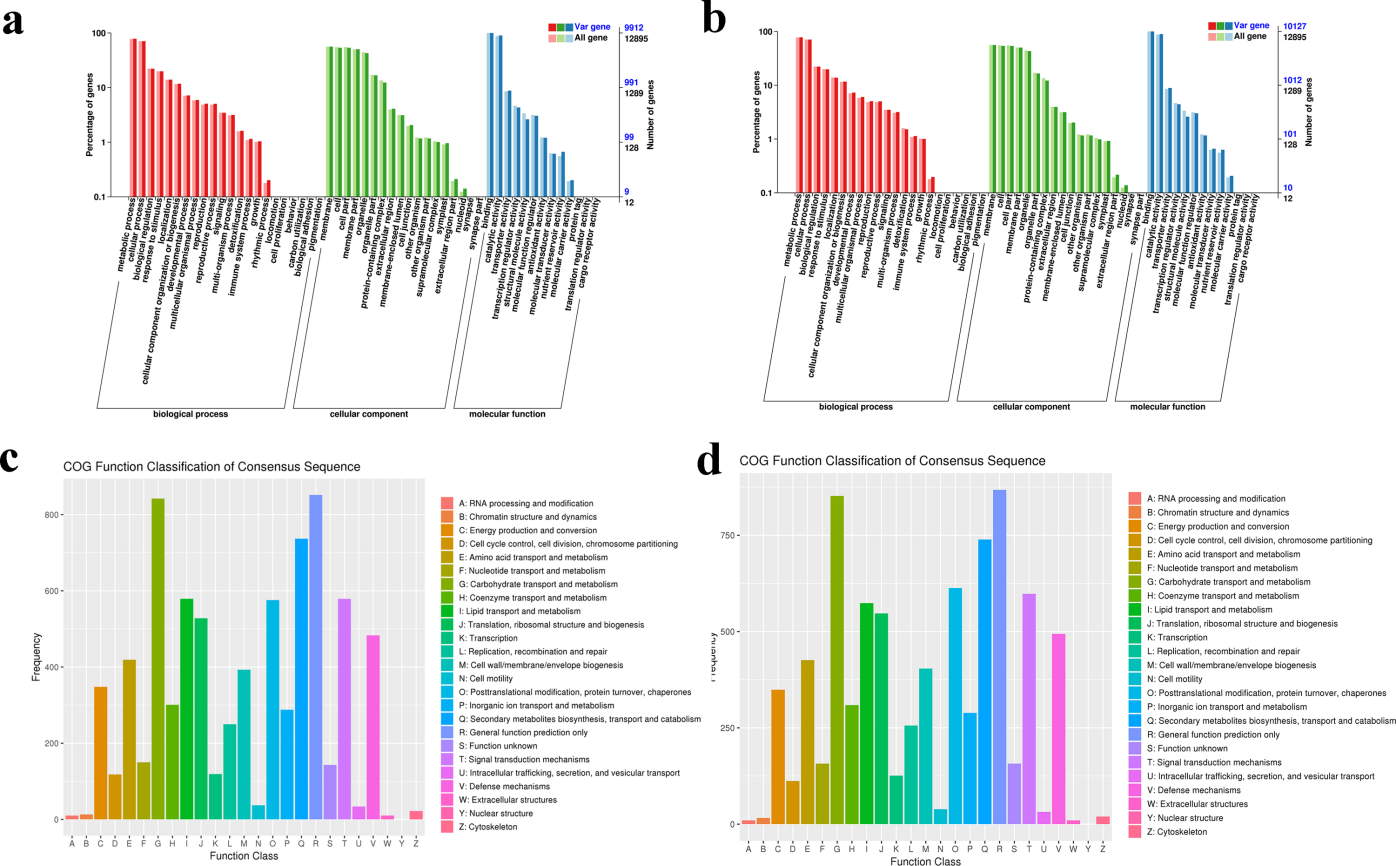
GO and COG annotation clustering of variant genes. (A) GO annotation clustering of variant genes in samples FD. (B) GO annotation clustering of variant genes in samples YP1. (C) COG annotated taxonomic map of variant genes of sample FD. (D) COG annotated taxonomic map of variant genes of sample YP1. The Abscissa of A and B is the content of GO classification, the left side of the ordinate is the percentage of the number of genes, and the right side is the number of genes. The Abscissa of C and D is the content of COG classification, and the ordinate is the number of genes. In different functional classes, the proportion of genes reflects the metabolic or physiological bias in the corresponding period and environment, which can be scientifically explained in combination with the distribution of research objects in each functional class.

Variations in the CDS region may cause changes in gene function, the variation genes of FD and YP1 were compared with GO, COG functional database, and these gene annotations were obtained to analyze gene function. In the GO annotation map of the variant genes of FD and YP1 (Figs. 3A, 3B), they are divided into three categories, namely biological process, cellular component and molecular function, in which the variant genes of FD and YP1 are expressed in 16 terms of biological process, of which FD annotated 3,049 GO items, 7,771 genes, YP1 annotated 3,061 items and 7,881 genes. The significantly rich items of FD and YP1 are biological process (GO: 0008150), FD and YP1 have 1,752 gene mutations, cellular process (GO:0009987), FD and YP1 have 1,328 gene variation. The variant genes of FD and YP1 are expressed in 16 terms of cellular component, of which FD annotates 607 GO items, 7,599 genes, YP1 annotates 605 items and 7,788 genes. The most significant rich item of FD and YP1 is integral component of membrane (GO:0016021), which has 6,148 gene mutations. The mutant genes of FD and YP1 were expressed in 10 terms of molecular function, including 1,139 GO entries and 14,339 genes annotated by FD and 1,147 items and 18,585 genes by YP1. The most significant rich item of FD and YP1 was ATP binding (GO:0005524), which had 2,639 gene variations.

**Table 3:** Classified statistics of differential genes produced by various variants.

**Smaple ID**	**Genes with non-synonymous SNP**	**Genes with InDel**	**Genes with SV**
FD	23,338	7,796	4,907
YP1	24,631	8,132	2,881

COG database can reflect the lineal homologous classification of gene products. COG functional classification map analysis showed that FD and YP1 were annotated in 24 categories (Figs. 3C, 3D), of which the richer categories were carbohydrate transport and metabolism, general function prediction only, secondly metabolites biosynthesis and transport and catabolism, and the least annotated category was RNA processing and modification.

### Mining of variant genes of crocin and geniposide

Gardenia fruit will accumulate a large number of chemicals in the process of development, among which geniposide and crocin are the main medicinal components. In order to better explore the related variant genes of crocin and geniposide in cultivar FD and wild species YP1, the structural genes in the biosynthesis pathway of crocin and geniposide (Figs. S5A, S5B) were identified by KEGG. The structural gene GjCCD4a (Gj9A597T69) of crocin was annotated on Carotenoid biosynthesis (ko00906) (Table S5). In this pathway, FD annotated 91 genes, including 79 variant genes, YP1 annotated 91 genes and 78 variant genes. The differential genes were annotated by Gj11A235T45, GjALDH2C3 (Gj9A24T70) in Phenylpropanoid biosynthesis (ko00940). FD annotated a total of 566 genes and 456 mutant genes. YP1 annotated 566 genes, 456 mutant genes, GjUGT94E13 (Gj9P1027T10) annotated on two KEGG pathways, Flavonoid biosynthesis (ko00941), Flavone and flavonol biosynthesis (ko00944), FD annotated 174 genes on Flavonoid biosynthesis, 134 genes were variant genes, YP1 annotated 174 genes, 136 genes were variant genes, FD annotated 45 genes on Flavone and flavonol biosynthesis, of which 37 genes were variant genes, YP1 annotated 40 genes, and 45 genes were variant genes.

The key structural genes GES (Gj7A350T74), G10H (Gj9A674T167), 10-HGO (Gj9P312T6) and IS (Gj9A1001T119) for geniposide synthesis were identified by TBtools comparison, in which GES (Gj7A350T74) was not annotated on the KEGG pathway, G10H (Gj9A674T167) was annotated on monoterpenoid biosynthesis (ko00902), a total of 70 genes were annotated, FD annotated 59 mutant genes and YP1 annotated 58 mutant genes. 10-HGO (Gj9P312T6) annotates 96, 355, 219, 121 genes in fatty acid degradation (ko00071), carbon metabolism (ko00071), glycolysis/gluconeogenesis (ko00010), Tyrosine metabolism (ko00350), FD annotates 79 mutant genes on the fatty acid degradation (ko00071) pathway, YP1 annotates 80 variant genes, FD annotates 292 variant genes on the carbon metabolism (ko00071) pathway, YP1 annotates 291 variant genes on the glycolysis/gluconeogenesis (ko00010) pathway. FD annotated 176variant genes and YP1 annotated 173variant genes. In the Tyrosine metabolism (ko00350) pathway, FD annotated 97 variant genes, YP1 annotated 96 variant genes, and IS (Gj9A1001T119) did not annotate the synthetic pathway of KEGG.

## Discussion

*G. jasminoides* is widely distributed in the tropics of Southeast Asia, but it has been cultivated in China for at least 1000 years. Gardenia population is rich in genetic diversity, and there is a close relationship between geographical distance and genetic distance between populations (Han et al., 2007; Yang et al., 2016). At present, it is generally believed that there is genetic variation among different populations of gardenia in China, and it has adapted to different living environments in the long-term process of cultivation and domestication (Mei et al., 2015). However, the differences and diversity between cultivated and wild populations have not been well explored.

In this study, a total of 39,406,575 clean reads were obtained from one wild and cultivated species, and the average depth of each sample was 10X. A large number of variations such as SNP, InDel, SV and CNV were detected. Compared with other mutations, SNP and InDel have more abundant genetic variation and can better represent the genetic information of the whole genome of gardenia. SNP and InDel variation analysis have been widely used in the fields of population genetic structure, genetic diversity analysis and functional gene mining of non-model organisms (Chen et al., 2021; Giudice, Bazakos & Vassiliou, 2022). 3,087,176 SNP variants were detected in cultivar gardenia FD, and 3,241,416 SNP variants were detected in YP1. The same was true of the number of SNP variations in wild species YP1 than in cultivated species FD1, InDel. It can be seen that wild species YP1 has higher genetic diversity, and in different *Brassica napus*, it is also found that winter rape has higher genetic diversity because the density of SNP and InDel is higher than that of spring rape (Wu et al., 2019). The most abundant type of SNP variation in sample YP1 and FD was the transformation type, and there was almost no difference between the conversion type of FD and the subversion type of SNP and YP1, especially the SNP ratio of heterozygous type of YP1 was much higher than that of FD, which indicated that YP1 had higher heterozygosity than FD and had higher homology with gardenia material with reference genome, while the cultivated species FD had more homozygous SNP than wild species YP1. It is reported in different varieties of *Camellia sinensis* that the ratio of non-synonymous mutation to synonymous mutation of InDel variation is almost the same as that of long-term interspecific hybridization. It is inferred that to a large extent, these two samples and the reference genome of gardenia do not come from the same region (An et al., 2020). Transition (Ti) and transversion (Tv) are two types of SNP variants, and the proportion between them is related to the evolution of the species (Mursyidin et al., 2022), the proportion of Ti/Tv between the two samples is very close, which does not rule out the common origin and ancestor of cultivated species FD and wild species YP1. The number of the main mutation types of FD was also less than that of YP1, and the variations of the two samples were mainly concentrated in a large number of frame shift mutations in the intergenic region. Generally speaking, the variation of FD of cultivated species is relatively conservative, which has lost a lot of genetic diversity while having more stable quality, which is similar to the reported results of pear, cotton and wheat (Li et al., 2021a; Li et al., 2021b; Hussain et al., 2022).

This study also explored the variation genes of geniposide and crocin, which are the main medicinal substances in gardenia fruit. The mining of variation genes related to medicinal substances is helpful to the improvement and application of gardenia genetic germplasm. The key genes in geniposide synthesis pathway are mainly annotated on five pathways, of which Tyrosine metabolism (ko00350) and Monoterpenoid biosynthesis (ko00902) have been shown to be closely related to geniposide synthesis in plants (Pan et al., 2021). Tyrosine acts as the precursor of many specialized metabolites, which have a variety of physiological functions. Some plant natural products derived from Tyrosine are also used in human medicine and nutrition, and in some Monoterpenoid plant products, they are used as effective drugs for a variety of diseases (Schenck & Maeda, 2018; Nguyen et al., 2022). Not only that fatty acid degradation (ko00071), carbon metabolism (ko00071), glycolysis/gluconeogenesis (ko00010) are also synthetic precursors of many metabolites (Newman et al., 2008; Lin et al., 2019; Toleco et al., 2020). The variant genes with differences in these pathways need to be further explored in the future. One of the important synthetic precursors of crocin is carotenoid. It is reported that carotenoid biosynthesis pathway has a great influence on the synthesis of crocin in gardenia (Shen et al., 2022), and 30 DEGs have been annotated on the carotenoid biosynthesis KEGG pathway of gardenia (Pan et al., 2021). In addition to carotenoid biosynthesis pathway, flavonoid biosynthesis (ko00941) and flavone and flavonol biosynthesis (ko00944) are important secondary metabolites that exist widely in plants, which not only play an important role in plant growth and development, but also have outstanding applications in food and medicine. The KEGG pathways annotated by CCD4a, ALDH2C3, UGT74F8, UGT94E13 of crocin structural genes are all involved in regulating pigment metabolism. This is also the main reason for the color change of gardenia after ripening (Sommano et al., 2020; Liu et al., 2021; Zhang et al., 2023).

With the intensive artificial selection in pursuit of better quality and higher yield, the cultivated germplasm resources have lost a lot of genetic diversity. It has been reported that in order to accelerate the biological research and genetic improvement of tomato, pan-genomic comparison was carried out between cultivated and wild species of tomato to reveal SVs (Li et al., 2023). The number of samples selected in this study is small, and more wild and cultivated species of *G. jasminoides* have not been analyzed, so the genetic difference between samples is not significant. In the future, more germplasm resources are needed to explore the genetic diversity and origin of wild gardenia species, to establish a molecular marker system for the identification of different gardenia germplasm, and further functional characterization of the variation can better understand the genetic basis of the differences between cultivated species and their wild relatives.

## Conclusions

The whole genome of one wild and one cultivated species of gardenia was resequenced, and a large number of SNPs, InDels, CNVs and SVs mutations were detected. SNP and InDel variation are important tools for studying genomic diversity and genome-based breeding, which have extensive application prospects and important practical value. This study have rapidly expanded our understanding of genetic variation of medicinal plants and provided rich resources for genetic research. KEGG significant enrichment analysis showed that the richness of metabolic pathways proved the possible role of differential genes in important biological and metabolic pathways. It was found that the related genes in geniposide and crocin biosynthesis pathway may have mutations, and the corresponding molecular markers can be developed in follow-up research to mine excellent genes for further verification. The extensive variation between wild and cultivated species provided in this study provides a good way to make further use of the genetic diversity of wild gardenia species for gene-based breeding, and also has a certain guiding significance for the evolution and classification of *G. jasminoides*.

## Supplemental Information

10.7717/peerj.16056/supp-1 Supplemental Information 1 Venn diagram of SNP statistics between samples The venn statistics of the number of variation sites only considered whether the position was the same (the starting position of the Indel), not the genotype.

10.7717/peerj.16056/supp-2 Supplemental Information 2 Sample chromosome coverage depth distribution map Note: The abscissa is the chromosome position, and the ordinate is the value obtained by logarithm (log2) of the coverage depth of the corresponding position on the chromosome. a.the sample of FD,b.the sample of YP1

10.7717/peerj.16056/supp-3 Supplemental Information 3 SNP mutation distribution map

10.7717/peerj.16056/supp-4 Supplemental Information 4 InDel length distribution map of whole genome and coding region Note: the ordinate indicates the length of InDel, the Insertion is greater than 0, the Deletion is less than 0, and the abscissa indicates the corresponding quantity.The red is the sample of FD, the blue is YP1.

10.7717/peerj.16056/supp-5 Supplemental Information 5 Carotenoidand Geniposide biosynthesis a. Carotenoid biosynthesis pathway. b. Geniposide biosynthesis pathway

10.7717/peerj.16056/supp-6 Supplemental Information 6 Statistical table of sample coverage depth and coverage ratio Average depth: the average coverage depth of the sample; the last three columns represent the proportion of bases with coverage depth at or above a given depth to the total base number of the reference genome, which are 1x, 5x and 10x, respectively.

10.7717/peerj.16056/supp-7 Supplemental Information 7 The table of SNP mutation

10.7717/peerj.16056/supp-8 Supplemental Information 8 InDel statistics of whole genome and coding region CDS: InDel statistics of coding region; Genome: genome-wide InDel statistics; Insertion: number of inserts detected; Deletion: number of deletions detected; Het: number of heterozygous InDel; Homo: number of homozygous InDel; Total: total number of InDel detected (excluding duplicates).

10.7717/peerj.16056/supp-9 Supplemental Information 9 SV quantity statistics SV: total number of structural variation; INS: number of insertion type variation; DEL: number of deletion type variation; INV: number of inversion type variation; DUP: number of repetitive type variation; TRA: number of chromosome translocation type variation.

10.7717/peerj.16056/supp-10 Supplemental Information 10 Variant gene of Carotenoid and Geniposide biosynthesis

## Additional Information and Declarations

### Data Availability

The following information was supplied regarding data availability: The data is available in the Supplemental Files and at GenBank: PRJNA986953.
