# Supplementary figures and images for: Whole-genome resequencing analysis of the medicinal plant Gardenia jasminoides
Source: PeerJ. 2023 Sep 18;11:e16056. doi: 10.7717/peerj.16056 (PMC10512932; doi:10.7717/peerj.16056)

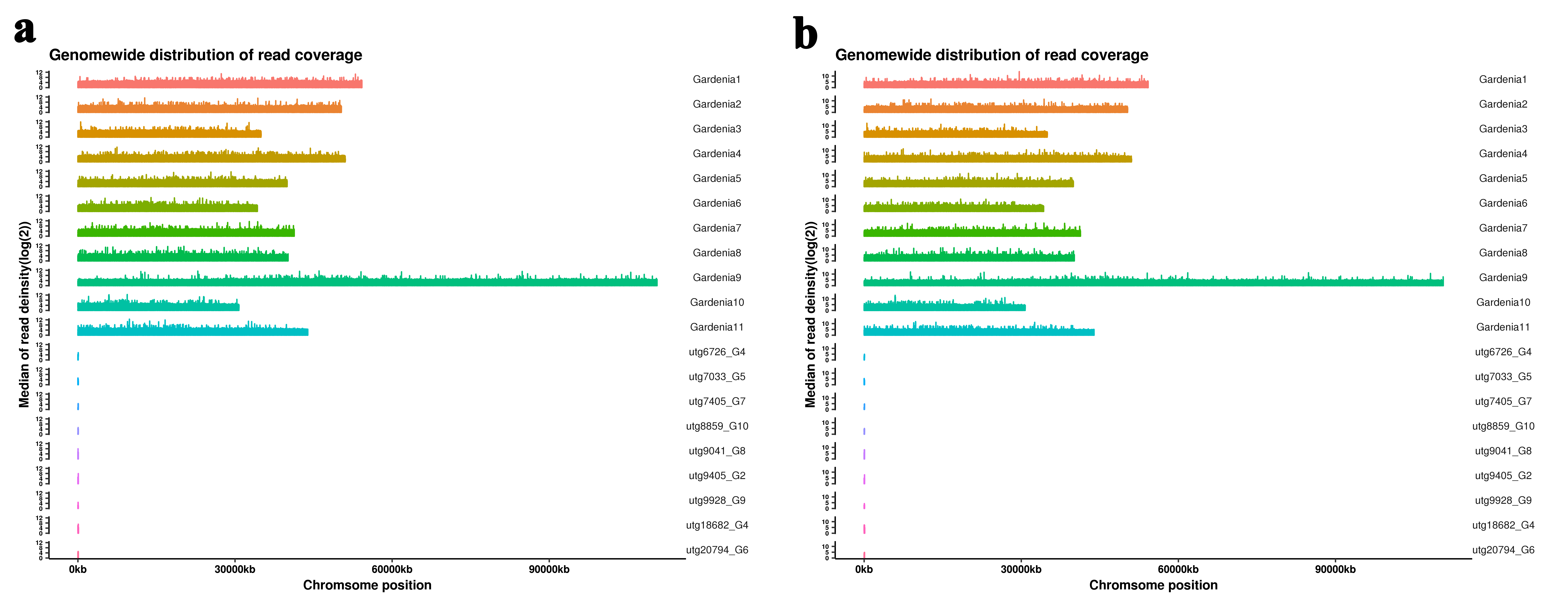

Supplement: Supplemental Information 1 — The venn statistics of the number of variation sites only considered whether the position was the same (the starting position of the Indel), not the genotype. [file peerj-11-16056-s001.png]

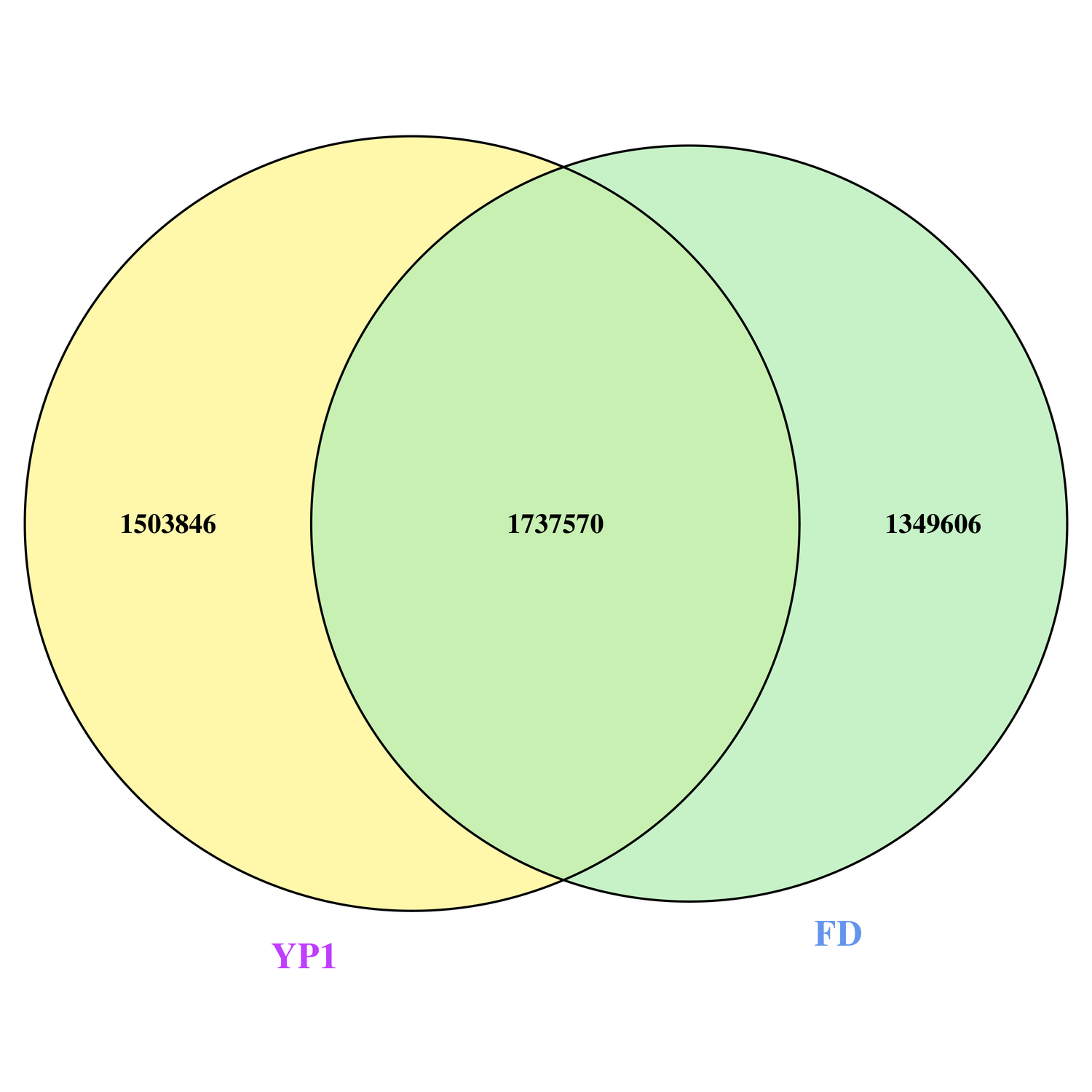

Supplement: Supplemental Information 2 — Note: The abscissa is the chromosome position, and the ordinate is the value obtained by logarithm (log2) of the coverage depth of the corresponding position on the chromosome. a.the sample of FD,b.the sample of YP1 [file peerj-11-16056-s002.png]

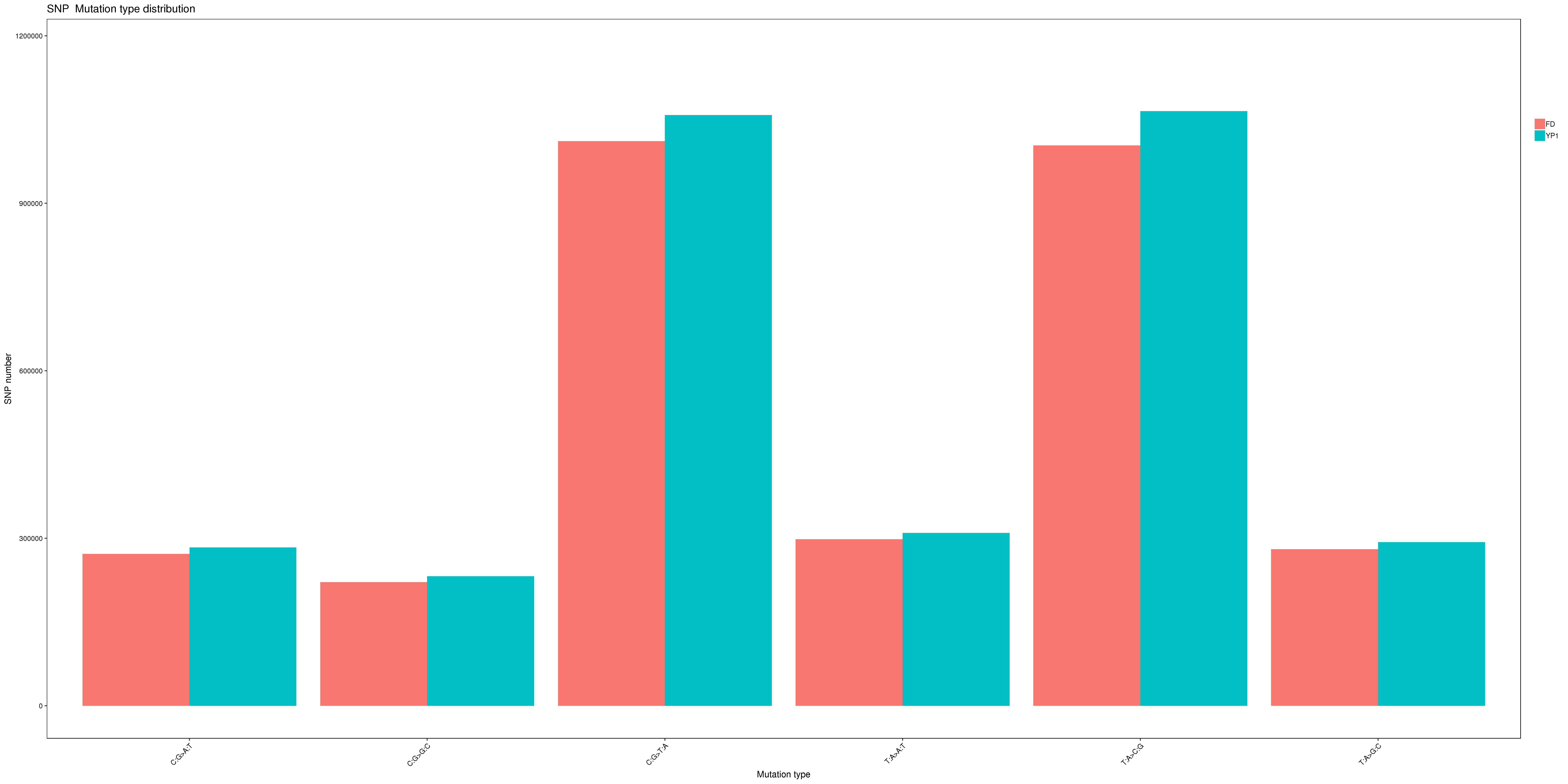

Supplement: Supplemental Information 3 [file peerj-11-16056-s003.png]

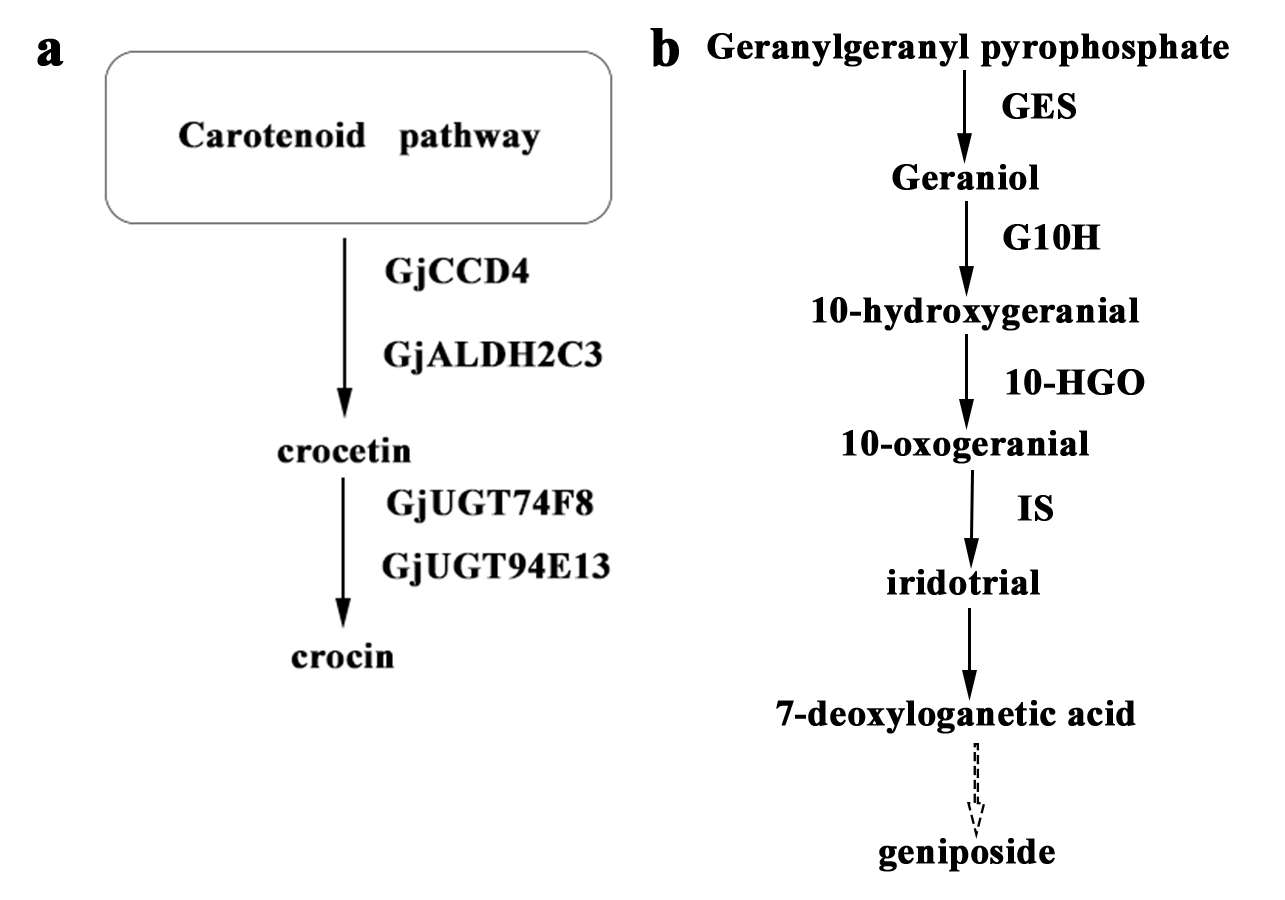

Supplement: Supplemental Information 5 — a. Carotenoid biosynthesis pathway. b. Geniposide biosynthesis pathway [file peerj-11-16056-s005.png]
