# Supplementary material for: Whole-genome resequencing analysis of the medicinal plant Gardenia jasminoides
Source: PeerJ. 2023 Sep 18;11:e16056. doi: 10.7717/peerj.16056 (PMC10512932; doi:10.7717/peerj.16056)
Supplement: Supplemental Information 6 — Average depth: the average coverage depth of the sample; the last three columns represent the proportion of bases with coverage depth at or above a given depth to the total base number of the reference genome, which are 1x, 5x and 10x, respectively. [file peerj-11-16056-s006.docx]

Table S1 Statistical table of sample coverage depth and coverage ratio

| **Sample ID** | **Average depth** | **Coverage ratio 1X(%)** | **Coverage ratio 5X(%)** | **Coverage ratio 10X(%)** |
| --- | --- | --- | --- | --- |
| FD | 11 | 85.02 | 71.13 | 46.02 |
| YP1 | 9 | 86.84 | 67.26 | 35.96 |

Average depth: the average coverage depth of the sample; the last three columns represent the proportion of bases with coverage depth at or above a given depth to the total base number of the reference genome, which are 1x, 5x and 10x, respectively.
