# Supplementary material for: Whole-genome resequencing analysis of the medicinal plant Gardenia jasminoides
Source: PeerJ. 2023 Sep 18;11:e16056. doi: 10.7717/peerj.16056 (PMC10512932; doi:10.7717/peerj.16056)
Supplement: Supplemental Information 7 [file peerj-11-16056-s007.docx]

Table S2 The table of SNP mutation

| **Sample ID** | **SNPnumber** | **Transition** | **Transversion** | **Ti/Tv** | **Heterozygosity** | **Homozygosity** | **Het-ratio** |
| --- | --- | --- | --- | --- | --- | --- | --- |
| FD | 3087176 | 2015058 | 1072118 | 1.87 | 1532772 | 1554404 | 49.64% |
| YP1 | 3241416 | 2123011 | 1118405 | 1.89 | 2174312 | 1067104 | 67.07% |
