# Supplementary material for: Whole-genome resequencing analysis of the medicinal plant Gardenia jasminoides
Source: PeerJ. 2023 Sep 18;11:e16056. doi: 10.7717/peerj.16056 (PMC10512932; doi:10.7717/peerj.16056)
Supplement: Supplemental Information 8 — CDS: InDel statistics of coding region; Genome: genome-wide InDel statistics; Insertion: number of inserts detected; Deletion: number of deletions detected; Het: number of heterozygous InDel; Homo: number of homozygous InDel; Total: total number of InDel detected (excluding duplicates). [file peerj-11-16056-s008.docx]

Table S3 InDel statistics of whole genome and coding region

| **Sample** | **CDS-Insertion** | **CDS-Deletion** | **CDS-Homo** | **CDS-Het** | **CDS-Total** | **Genome-Insertion** | **Genome-Deletion** | **Genome-Homo** | **Genome-Het** | **Genome-Total** |
| --- | --- | --- | --- | --- | --- | --- | --- | --- | --- | --- |
| FD | 5839 | 4431 | 6075 | 4195 | 10270 | 310024 | 248742 | 340686 | 218080 | 558766 |
| YP1 | 5937 | 4815 | 4804 | 5948 | 10752 | 295778 | 244803 | 251829 | 288752 | 540581 |
| Total | 7722 | 6823 | -- | -- | 14545 | 422343 | 368888 | -- | -- | 791231 |

CDS: InDel statistics of coding region; Genome: genome-wide InDel statistics; Insertion: number of inserts detected; Deletion: number of deletions detected; Het: number of heterozygous InDel; Homo: number of homozygous InDel; Total: total number of InDel detected (excluding duplicates).
