# Supplementary material for: Whole-genome resequencing analysis of the medicinal plant Gardenia jasminoides
Source: PeerJ. 2023 Sep 18;11:e16056. doi: 10.7717/peerj.16056 (PMC10512932; doi:10.7717/peerj.16056)
Supplement: Supplemental Information 9 — SV: total number of structural variation; INS: number of insertion type variation; DEL: number of deletion type variation; INV: number of inversion type variation; DUP: number of repetitive type variation; TRA: number of chromosome translocation type variation. [file peerj-11-16056-s009.docx]

Table S4 SV quantity statistics table

| **SMAPLE ID** | **SV** | **INS** | **DEL** | **INV** | **DUP** | **TRA** |
| --- | --- | --- | --- | --- | --- | --- |
| FD | 11543 | 1095 | 6145 | 269 | 460 | 3574 |
| YP1 | 7634 | 561 | 4297 | 148 | 250 | 2378 |

SV: total number of structural variation; INS: number of insertion type variation; DEL:number of deletion type variation; INV: number of inversion type variation; DUP: number of repetitive type variation; TRA: number of chromosome translocation type variation.
