# Supplementary material for: Whole-genome resequencing analysis of the medicinal plant Gardenia jasminoides
Source: PeerJ. 2023 Sep 18;11:e16056. doi: 10.7717/peerj.16056 (PMC10512932; doi:10.7717/peerj.16056)
Supplement: Supplemental Information 10 [file peerj-11-16056-s010.docx]

| Pathway | Ko ID | Variant gene | gene | Variant gene all | Gene all |
| --- | --- | --- | --- | --- | --- |
| Carotenoid biosynthesis | ko00906 | 79 | 91 | 7578 | 9849 |
| Carotenoid biosynthesis | ko00906 | 78 | 91 | 7760 | 9849 |
| Phenylpropanoid biosynthesis | ko00940 | 456 | 566 | 7578 | 9849 |
| Phenylpropanoid biosynthesis | ko00940 | 449 | 566 | 7760 | 9849 |
| Flavonoid biosynthesis | ko00941 | 134 | 174 | 7578 | 9849 |
| Flavone and flavonol biosynthesis | ko00944 | 37 | 45 | 7578 | 9849 |
| Flavone and flavonol biosynthesis | ko00944 | 40 | 45 | 7760 | 9849 |
| Flavonoid biosynthesis | ko00941 | 136 | 174 | 7760 | 9849 |

Table S5.Variant gene of Carotenoid and Geniposide biosynthesis
